# Editorial: Magnetoencephalography (MEG) in Epilepsy and Neurosurgery

**DOI:** 10.3389/fnhum.2022.873153

**Published:** 2022-03-14

**Authors:** Vahe Poghosyan, Stefan Rampp, Zhong Irene Wang

**Affiliations:** ^1^Department of Neurophysiology, National Neuroscience Institute, King Fahad Medical City, Riyadh, Saudi Arabia; ^2^Department of Neurosurgery, University Hospital Erlangen, Erlangen, Germany; ^3^Department of Neurosurgery, University Hospital Halle (Saale), Halle (Saale), Germany; ^4^Charles Shor Epilepsy Center, Neurological Institute, Cleveland Clinic, Cleveland, OH, United States

**Keywords:** magnetoencephalography (MEG), magnetic source imaging (MSI), presurgical evaluation, epilepsy surgery, drug-resistant epilepsy (DRE)

Around 30% of all patients with epilepsy are resistant to anti-seizure medications (Kalilani et al., 2018), leading to significant morbidity and mortality. Epilepsy surgery, involving removal of the epileptogenic zone, is the safest and most effective treatment for this group of patients (Ryvlin et al., 2014). Favorable outcome of epilepsy surgery depends critically upon accurate delineation of the epileptogenic zone (Najm et al., 2013). Magnetoencephalography (MEG) has been shown to provide clinically significant information to this end (Knowlton et al., 2008; Murakami et al., 2016; Rampp et al., 2019). However, it is still underutilized and is not included in the standard of care in a large majority of medical centers (Shiraishi et al., 2012; Mouthaan et al., 2016; Bagic and Burgess, 2020).

In this Research Topic, we have assembled original research papers, case reports, methods and review articles by eminent clinicians and researchers to emphasize the unique added value of MEG in presurgical evaluation of patients with epilepsy, in order to promote its wider utilization in clinical practice. The articles reveal novel methods and procedures that can improve the yield of MEG in epilepsy surgery, and describe significant clinical scenarios, where MEG, with advanced methods for analysis, can be utilized to achieve favorable surgical outcome.

Papadelis et al. present a case of a patient who suffered from drug-resistant epilepsy for 16 years. An extensive presurgical evaluation of the patient, using MEG and other advanced and well-established techniques, accurately identified the epileptogenic zone in the right anterior insula. Ablation of the identified brain region with laser interstitial thermal therapy rendered the patient seizure-free. The MEG source localization method used in this case has identified the epileptogenic zone with an accuracy of approximately 12 mm, which is consistent with many previous reports. Of note, the magnetic resonance imaging (MRI) study was initially reported as nonlesional. However, MEG and electroencephalography-directed review of the MRI data revealed an abnormality in the right anterior insula that was suggestive of focal cortical dysplasia. In general, MEG-directed review of MRI can identify previously undetected epileptogenic abnormalities in up to 50% of patients (Kharkar and Knowlton, 2015). Therefore, a re-evaluation of “non-lesional” MRIs following positive MEG findings is recommended.

In another case report, van Heumen et al. present a case of an 8-year old girl with drug-resistant epilepsy who underwent presurgical evaluation including ictal MEG, and resective surgery that led to seizure freedom. Two points are of significance in this paper. First, while interictal MEG is the current standard for routine clinical MEG studies (Bagic et al., 2011), this and other recent studies have demonstrated the feasibility and clinical usefulness of ictal MEG recordings. Unfortunately, currently, there is no guideline or consensus concerning analysis and interpretation of ictal MEG data. Thus, studies use a variety of methods and procedures to these ends (Alkawadri et al., 2018). In this case, van Heumen et al. successfully delineated the seizure onset zone based on distributed source imaging (unconstrained volumetric dSPM) of ten averaged peaks of rhythmic alpha oscillations at the ictal onset. Second, this study provides novel evidence suggesting that broadband aperiodic fluctuations of ongoing brain activity, which are traditionally discarded as “brain noise,” contain useful information that can help in localizing the seizure onset zone. Recognition of such measures, which do not require definition of discrete epileptic events (spikes, seizure onset etc.) or frequency bands of interest by an expert to delineate the epileptogenic zone, can be highly valuable in presurgical evaluation of patients with epilepsy. They may be more objective (operator-independent), applicable to a wider patient population (e.g., in cases with no interictal discharges in MEG data) and faster to obtain (no need for visual analysis of large datasets).

Xu et al. review current literature concerning the use of MEG resting-state functional connectivity in presurgical evaluation of patients with epilepsy. They briefly survey the prevalent methods for MEG source imaging and source-based connectivity metrics, and the open-source research tools that implement them. The paper describes available evidence on altered resting-state functional connectivity patterns in various types of epilepsy, highlighting the potential value of connectivity metrics in predicting the postsurgical seizure outcome. Similar to the measures of aperiodic neural activity investigated by van Heumen et al. estimation of the epileptogenic zone from the resting-state functional connectivity measures, without the need to mark discrete epileptic events, can be beneficial in clinical practice.

Cai et al. provide clinical validation of the Champagne algorithm (Cai et al., 2021) for the localization of interictal MEG spikes. Champagne is a robust empirical Bayesian source reconstruction algorithm that previously has been successfully used in a number of other clinical and research applications. In this study, authors compared its performance with equivalent current dipole fit, which is the current standard for MEG spike source localization in clinical practice, in three common scenarios involving a cohort of 16 patients. The results show that the Champagne algorithm is equivalent to dipole fit in relatively straightforward cases, and may outperform it in more complex localization scenarios. These suggest that Champagne may be a reliable and more automatic alternative to the traditional dipole fitting methods for the localization of interictal MEG spikes.

A clinical MEG study, for the localization of the epileptogenic zone or functional cortical mapping, can be performed in an inpatient or outpatient setting. Watkins et al. conducted a detailed retrospective institutional review of inpatient pediatric MEG studies to identify the clinical circumstances where an inpatient versus outpatient MEG should be considered. They have identified five indications, where an inpatient MEG study can lead to timely and improved decision-making, providing a more efficient and better overall patient care.

The articles in this Research Topic reiterate the clinical usefulness and added value of MEG in epilepsy presurgical evaluation, and show that clinical MEG is a dynamic field with invariably evolving methods and applications. We anticipate increased utilization of MEG in neurosurgery as well as in other aspects of epilepsy management, and hope that this Research Topic will motivate researchers and clinicians to develop and explore new methods and clinical applications.

## Author Contributions

VP wrote the first draft of the manuscript. All authors contributed to manuscript revision, read, and approved the submitted version.

## Conflict of Interest

The authors declare that the research was conducted in the absence of any commercial or financial relationships that could be construed as a potential conflict of interest.

## Publisher's Note

All claims expressed in this article are solely those of the authors and do not necessarily represent those of their affiliated organizations, or those of the publisher, the editors and the reviewers. Any product that may be evaluated in this article, or claim that may be made by its manufacturer, is not guaranteed or endorsed by the publisher.
